# Supplementary material for: Using a natural capital risk register to support the funding of seagrass habitat enhancement in Plymouth Sound
Source: PeerJ. 2024 Oct 28;12:e17969. doi: 10.7717/peerj.17969 (PMC11526789; doi:10.7717/peerj.17969)
Supplement: Supplemental Information 1 [file peerj-12-17969-s001.docx]

**Supplementary Materials 1 – Input Data Products**

The following input data products that underpin the Plymouth Sound Seagrass Asset and Risk Register. Data sources and methodologies for each data product are provided with references in the sections below.

Contents

[List of Abbreviations 2](#_Toc169263448)

[Composite Habitat Map 3](#_Toc169263449)

[The Asset Benefit Matrix 6](#_Toc169263450)

[The Condition of Seagrass Habitat at the Feature Level. 13](#_Toc169263451)

[The Condition of Seagrass Habitat at the individual Seagrass Bed level 22](#_Toc169263452)

[Habitat Suitability Data 35](#_Toc169263453)

# List of Abbreviations

AMS – Advanced Mooring System

C – Carbon

CBD – Convention on Biological Diversity

EC – European Council

ES – Ecosystem Services

EU – European Union

EUNIS – The European Nature Information System

IFCA – Inshore Fisheries Conservation Authority

JNCC – Joint Nature Conservation Committee

LRC – Likely Relative Condition

MCS – Marine Conservation Society

MCZ – Marine Conservation Zone

MEA – Millennium Ecosystem Assessment

MMO – Marine Management Organisation

MPA – Marine Protected Area

MSFD – Marine Strategy Framework Directive

NE – Natural England

NEA – National Ecosystem Assessment

OCT – Ocean Conservation Trust

ReMEDIES - Reducing and Mitigating Erosion and Disturbance Impacts affecting the Seabed.

SAC – Special Area of Conservation

UoP – University of Plymouth

# Composite Habitat Map

**Method**

The seagrass habitats present within the Plymouth Sound area, up to mean high water, were derived from best available habitat map data available for the region (Table 1). A composite habitat map was generated that combined these data sets, prioritizing by MESH confidence, age of data and EUNIS level (Figure 1). Additional data describing specific seagrass beds were provided by Ollie Thomas and Mark Parry (OCT).

The extent (km²) of each seagrass bed habitat occurring within the Plymouth Sound and Estuaries SAC were calculated from the composite habitat map, in ESRI ArcGIS Pro (Figure 2).

Table 1: Data sources for the composite habitat map

| **Dataset** | **Source** | **Link** |
| --- | --- | --- |
| Habitat data from survey | Various sources, via EMODnet seabed habitats: GB000228, GB000229, GB000283, GB000335, GB001069,  GB001070, GB001072,  GB100072, GB100206,  GB100207 | https://emodnet.ec.europa.eu/en/seabed-habitats |
| Modelled habitat data | UKSeaMap (2018 v2) | https://hub.jncc.gov.uk/assets/202874e5-0446-4ba7-8323-24462077561e |
| Additional seagrass bed boundary data | Oliver Thomas PhD data, Thanckes Lake and Wilcove bed extents |  |
| Additional seagrass bed boundary data | Ocean Conservation Trust seagrass bed extents | https://oceanconservationtrust.org/ |

Figure 1: Composite Habitat map for all seabed habitat in the study area. Map made in ArcGIS Pro by the University of Plymouth. Habitat data obtained from UKSeaMap: JNCC (Open Government License, https://hub.jncc.gov.uk/assets/202874e5-0446-4ba7-8323-24462077561e) and EMODnet Seabed Habitats Initiative (emodnet-seabedhabitats.eu).

Figure 2: Map of all seagrass assets (intertidal and subtidal) within the study area. Map made in ArcGIS Pro by the University of Plymouth. Habitat data obtained from UKSeaMap: JNCC (Open Government License, https://hub.jncc.gov.uk/assets/202874e5-0446-4ba7-8323-24462077561e), EMODnet Seabed Habitats Initiative (emodnet-seabedhabitats.eu), the University of Plymouth and the Ocean Conservation Trust.

# The Asset Benefit Matrix

The Seagrass asset benefit matrix (Table 2) uses established matrices to define ecosystem services from UK seagrass habitats (Fletcher, Herbert and Saunders, 2012; Potts *et al.*, 2014; Saunders *et al.*, 2015). Additional literature in the list below was used to determine the results of the Seagrass Asset and Benefit Matrix.

Table 2: Contribution of seagrass habitat features within the study area to 5 key ES benefits: wild food, sea defense, clean water and sediments, healthy climate, tourism including recreation and nature watching in the study area (ES contributions reviewed from existing studies (Potts et al., 2014; Saunders et al., 2015; Rees et al., 2022). Cell shade indicates the scale of ecosystem service contribution (Black = significant ; dark grey = moderate; light grey = low; white = no or negligible). The number indicates the confidence in evidence available to assign ES provision (3 = UK-related, peer-reviewed literature; 2 = Grey or overseas literature; 1 = Expert opinion).

|  |  | Contribution to ES Good/Benefits | | | | |
| --- | --- | --- | --- | --- | --- | --- |
|  | | Provisioning Service | Regulating Services | | | Cultural Services |
| Seagrass Natural Capital Assets: Seagrass Habitat in the Plymouth Sound Estuaries and Coastal Area | | Food (wild food) | Clean water and sediments | Sea defence | Healthy climate | Tourism, nature watching and recreation |
| Intertidal Seagrass | | 3 | 1 | 1 | 1 | 1 |
| Subtidal Seagrass | | 3 | 2 | 1 | 2 | 1 |

| Scale of ecosystem service contribution relative to other features | |
| --- | --- |
| *#* | Significant contribution |
| *#* | Moderate |
| *#* | Low |
| *#* | No or negligible |
| *(Blank)* | Not assessed |

| Confidence in evidence available to assign ES provision |  |
| --- | --- |
| 3 | UK-related, peer-reviewed literature |
| 2 | Grey or overseas literature |
| 1 | Expert opinion |
| (Blank) | Not assessed |

**Main Reference List**

Potts T, Burdon D, Jackson E, Atkins J, Saunders J, Hastings E, Langmead O. 2014. Do marine protected areas deliver flows of ecosystem services to support human welfare? *Marine Policy* 44:139–148.

Saunders J, Potts T, Jackson E, Burdon D, Atkins JP, Hastings E, Langmead O, Fletcher S. 2015. Linking ecosystem services of marine protected areas to benefits in human wellbeing? In: *Coastal Zones Ecosystem Services*. Springer, 167–190.

Fletcher S, Herbert R, Saunders J. 2012.Description of the ecosystem services provided by broad-scale habitats and features of conservation importance that are likely to be protected by Marine Protected Areas in the Marine Conservation Zone Project area. *Available at* *https://www.researchgate.net/publication/269518514_Description_of_the_ecosystem_services_provided_by_broad-scale_habitats_and_features_of_conservation_importance_that_are_likely_to_be_protected_by_Marine_Protected_Areas_in_the_Marine_Conservation_Zone* (accessed July 26, 2023).

**Additional Literature References**

Ashley M, Rees S, Mullier T. 2021. *Natural Capital Asset and Risk Register to Inform Marine Site Management Plans and Implementation of Plymouth National Marine Park. Part One: Introduction to Natural Capital Assets and Ecosystem Service Benefits within Plymouth Sound, Estuaries and Coastal Area. Report compiled by staff at the University of Plymouth.*

Bertelli CM, Unsworth RKF. 2014. Protecting the hand that feeds us: Seagrass (Zostera marina) serves as commercial juvenile fish habitat. *Marine Pollution Bulletin* 83:425–429. DOI: https://doi.org/10.1016/j.marpolbul.2013.08.011.

Burrows MT, Kamenos NA, Hughes DJ, Stahl H, Howe JA, Tett P. 2014. *Assessment of carbon budgets and potential blue carbon stores in Scotland’s coastal and marine environment. Scottish Natural Heritage Commissioned Report No. 761.*

Christianen MJA, van Belzen J, Herman PMJ, van Katwijk MM, Lamers LPM, van Leent PJM, Bouma TJ. 2013. Low-Canopy Seagrass Beds Still Provide Important Coastal Protection Services. *PLOS ONE* 8:e62413. DOI: 10.1371/journal.pone.0062413.

Costanza R, d’Arge R, De Groot R, Farber S, Grasso M, Hannon B, Limburg K, Naeem S, O’neill R V, Paruelo J. 1997. The value of the world’s ecosystem services and natural capital. *nature* 387:253–260.

d’Avack EAS, Tyler-Walters H, Wilding CM, Garrard SL. 2022. Zostera (Zosterella) noltei beds in littoral muddy sand. In Tyler-Walters H. and Hiscock K. (eds) Marine Life Information Network: Biology and Sensitivity Key Information Reviews.

d’Avack EAS, Tyler-Walters H, Wilding CM, Garrard SL. 2022.Zostera (Zostera) marina beds on lower shore or infralittoral clean or muddy sand. In Tyler-Walters H. and Hiscock K. (eds) Marine Life Information Network: Biology and Sensitivity Key Information Reviews. *Available at* *https://www.marlin.ac.uk/habitat/detail/257* (accessed July 28, 2023).

de los Santos CB, Olivé I, Moreira M, Silva A, Freitas C, Araújo Luna R, Quental-Ferreira H, Martins M, Costa MM, Silva J, Cunha ME, Soares F, Pousão-Ferreira P, Santos R. 2020. Seagrass meadows improve inflowing water quality in aquaculture ponds. *Aquaculture* 528:735502. DOI: https://doi.org/10.1016/j.aquaculture.2020.735502.

do Amaral Camara Lima M, Bergamo TF, Ward RD, Joyce CB. 2023. A review of seagrass ecosystem services: providing nature-based solutions for a changing world. *Hydrobiologia* 850:2655–2670. DOI: 10.1007/S10750-023-05244-0/FIGURES/4.

do Amaral Camara Lima M, Ward RD, Joyce CB, Kauer K, Sepp K. 2022. Carbon stocks in southern England’s intertidal seagrass meadows. *Estuarine, Coastal and Shelf Science* 275:107947. DOI: https://doi.org/10.1016/j.ecss.2022.107947.

do Amaral Camara Lima M, Ward R, Joyce C. 2020. Environmental drivers of sediment carbon storage in temperate seagrass meadows. *Hydrobiologia* 847. DOI: 10.1007/s10750-019-04153-5.

Duarte CM, Middelburg JJ, Caraco N. 2005. Major role of marine vegetation on the oceanic carbon cycle. *Biogeosciences* 2:1–8. DOI: 10.5194/bg-2-1-2005.

Duarte CM, Kennedy H, Marbà N, Hendriks I. 2013. Assessing the capacity of seagrass meadows for carbon burial: Current limitations and future strategies. *Ocean & Coastal Management* 83:32–38. DOI: 10.1016/J.OCECOAMAN.2011.09.001.

EMODnet/EUSeaMap. 2019.European marine observation data network (EMODnet) seabed habitats project, Map Viewer, Download & Metadata Catalogue. *Available at* *https://emodnet.ec.europa.eu/en/seabed-habitats*

Fraser MW, Kendrick GA. 2017. Belowground stressors and long-term seagrass declines in a historically degraded seagrass ecosystem after improved water quality. *Scientific Reports* 7:14469. DOI: 10.1038/s41598-017-14044-1.

Green AE, Unsworth RKF, Chadwick MA, Jones PJS. 2021. Historical Analysis Exposes Catastrophic Seagrass Loss for the United Kingdom. *Frontiers in Plant Science* 12. DOI: 10.3389/fpls.2021.629962.

Green A, Chadwick MA, Jones PJS. 2018. Variability of UK seagrass sediment carbon: Implications for blue carbon estimates and marine conservation management. *PLOS ONE* 13:e0204431. DOI: 10.1371/journal.pone.0204431.

Gregg R, Elias JL, Alonso I, Crosher IE, Muto P, Morecroft MD. 2021. *Carbon storage and sequestration by habitat: a review of the evidence (second edition) Natural England Research Report NERR094.* Natural England, York.

Greiner JT, McGlathery KJ, Gunnell J, McKee BA. 2013. Seagrass Restoration Enhances “Blue Carbon” Sequestration in Coastal Waters. *PLOS ONE* 8:e72469. DOI: 10.1371/journal.pone.0072469.

Howard-Williams E. 2022. *Seagrass Natural Capital Assessment: Plymouth Sound and Estuaries SAC*.

Infantes E, Hoeks S, Adams MP, van der Heide T, van Katwijk MM, Bouma TJ. 2022. Seagrass roots strongly reduce cliff erosion rates in sandy sediments. *Marine Ecology Progress Series* 700:1–12.

JNCC. 2018. UKSeaMap 2018 (v2): Predictive mapping of seabed habitats in Uk waters.

Laffoley D, Grimsditch G. 2009. *The Management of Natural Coastal Carbon Sinks*.

Laing C, Hobson V. 2022. *Carbon accounts for eelgrass beds in the Fal and Helford Estuaries*.

Mcleod E, Chmura GL, Bouillon S, Salm R, Björk M, Duarte CM, Lovelock CE, Schlesinger WH, Silliman BR. 2011. A blueprint for blue carbon: toward an improved understanding of the role of vegetated coastal habitats in sequestering CO2. *Frontiers in Ecology and the Environment* 9:552–560. DOI: https://doi.org/10.1890/110004.

Miyajima T, Hori M, Hamaguchi M, Shimabukuro H, Adachi H, Yamano H, Nakaoka M. 2015. Geographic variability in organic carbon stock and accumulation rate in sediments of East and Southeast Asian seagrass meadows. *Global Biogeochemical Cycles* 29:397–415. DOI: https://doi.org/10.1002/2014GB004979.

Moore KA. 2004. Influence of seagrasses on water quality in shallow regions of the lower Chesapeake Bay. *Journal of Coastal Research*:162–178.

Mtwana Nordlund L, Koch EW, Barbier EB, Creed JC. 2016. Seagrass Ecosystem Services and Their Variability across Genera and Geographical Regions. *PLOS ONE* 11:e0163091. DOI: 10.1371/journal.pone.0163091.

Novak AB, Pelletier MC, Colarusso P, Simpson J, Gutierrez MN, Arias-Ortiz A, Charpentier M, Masque P, Vella P. 2020. Factors Influencing Carbon Stocks and Accumulation Rates in Eelgrass Meadows Across New England, USA. *Estuaries and Coasts* 43:2076–2091. DOI: 10.1007/s12237-020-00754-9.

Oreska MPJ, McGlathery KJ, Aoki LR, Berger AC, Berg P, Mullins L. 2020. The greenhouse gas offset potential from seagrass restoration. *Scientific Reports* 10:7325. DOI: 10.1038/s41598-020-64094-1.

Parker R, Benson L, Graves C, Kroger S, Vieira R. 2021. *Carbon stocks and accumulation analysis for Secretary of State (SoS) region, Cefas Report for Defra project ME5439, 42 pp.*

Poppe KL, Rybczyk JM. 2018. Carbon Sequestration in a Pacific Northwest Eelgrass (*Zostera marina*) Meadow. *Northwest Science* 92:80–91. DOI: 10.3955/046.092.0202.

POST. 2021. *POSTNOTE 0651 September 2021 Blue carbon. UK Parliament.*

Potouroglou M, Whitlock D, Milatovic L, MacKinnon G, Kennedy H, Diele K, Huxham M. 2021. The sediment carbon stocks of intertidal seagrass meadows in Scotland. *Estuarine, Coastal and Shelf Science* 258:107442. DOI: https://doi.org/10.1016/j.ecss.2021.107442.

Romero J, Pérez M, Mateo MA, Sala E. 1994. The belowground organs of the Mediterranean seagrass Posidonia oceanica as a biogeochemical sink. *Aquatic Botany* 47:13–19. DOI: https://doi.org/10.1016/0304-3770(94)90044-2.

Sandoval-Gil J, Alexandre A, Santos R, Camacho-Ibar VF. 2016. Nitrogen uptake and internal recycling in Zostera marina exposed to oyster farming: Eelgrass potential as a natural biofilter. *Estuaries and Coasts* 39:1694–1708.

Shilland R, Grimsditch G, Ahmed M, Bandeira S, Kennedy H, Potouroglou M, Huxham M. 2021. A question of standards: Adapting carbon and other PES markets to work for community seagrass conservation. *Marine Policy* 129:104574. DOI: 10.1016/J.MARPOL.2021.104574.

Spooner AM. 2015. Blue carbon sequestration potential in Zostera marina eelgrass beds of the K’ómoks Estuary, British Columbia. Canada -- British Columbia, CA: Royal Roads University (Canada) PP  - Canada -- British Columbia, CA.

Stafford R, Chamberlain B, Clavey L, Gillingham PK, McKain S, Morecroft MD, Morrison-Bell C, Watts O. 2021. *Nature-based Solutions for Climate Change in the UK: A Report by the British Ecological Society.* London, UK.

Unsworth RKF, Cullen LC, Pretty JN, Smith DJ, Bell JJ. 2010. Economic and subsistence values of the standing stocks of seagrass fisheries: Potential benefits of no-fishing marine protected area management. *Ocean & Coastal Management* 53:218–224. DOI: https://doi.org/10.1016/j.ocecoaman.2010.04.002.

Unsworth RKF, Cullen-Unsworth LC, Jones BLH, Lilley RJ. 2022. The planetary role of seagrass conservation. *Science* 377:609–613. DOI: doi:10.1126/science.abq6923.

Ward M, Cullen-Unsworth L, Geisler KG, Lilley R, Lynch J, Millington-Drake M, Pittman SJ, Smith A, Taylor S, Wedding LM, Wright R, Seddon N. 2023. DEVELOPING A UK SEAGRASS CARBON CODE JUNE 2023.

# The Condition of Seagrass Habitat at the Feature Level.

**Natural England Conservation Advice Packages**

Designated in 2005, Plymouth Sound and Tamar Estuaries (PSE) SAC (which is also the study site area for this project) covers an area of 6386.95 ha. The SAC contains a number of qualifying conservation features including intertidal and subtidal seagrass sub-features (*Z. marina and Z. noltei*). By reviewing the conservation advice package for Plymouth Sound and Estuaries SAC, found on Natural England’s designated sites online resource the conservation objectives for the seagrass features were collated (Table 3).

**Key Reference**:

Natural England. 2021.Plymouth Sound and Estuaries SAC. Feature Condition. *Available at* *https://designatedsites.naturalengland.org.uk/Marine/MarineFeatureCondition.aspx?SiteCode=UK0013111&SiteName=plymouth&SiteNameDisplay=Plymouth+Sound+and+Estuaries+SAC&countyCode=&responsiblePerson=&SeaArea=&IFCAArea=*

Table 3: Conservation objectives for relevant sub features on the Natural England online database.

| MPA | Feature | Subfeature | EUNIS | Condition | Relevant Management |
| --- | --- | --- | --- | --- | --- |
| Plymouth Sound and Estuaries SAC | Mudflats and sandflats not covered by seawater at low tide | Intertidal seagrass beds | A2.61 | Maintain | Cornwall side IFCA byelaws: Closed areas (European Marine Sites) No. 2. |
|  | Sandbanks which are slightly covered by sea water all the time | Subtidal seagrass beds | A5.53 | Restore | Cornwall IFCA byelaws:  Closed areas (European Marine Sites) No. 2.  Devon and Severn IFCA Byelaws: Mobile Fishing Permit Byelaw and Potting Permit Byelaw |

**Likely Relative Condition Modelling**

It is a statutory requirement that assessments of designated conservation features are carried out at least every 6 years. Data therefore has low temporal resolution. To support the assessment of condition for seagrass habitats across the Plymouth Sound Area a proxy approach was applied, using existing tools and data layers to determine habitat sensitivity to pressures, and activity data that may contribute to those pressures.

**Method**

Sensitivity information by EUNIS habitat was extracted from the Marine Evidence-based Sensitivity Assessment (MarESA) database (Tyler-Walters *et al.* 2022). MarESA compiles sensitivity information through a detailed literature review process of available evidence on the effects of pressures arising from human activities on marine habitats (Table 4). The assessments assign scores for habitat sensitivity as a combination of resistance and resilience to particular pressures. The scores allocated are: Not Sensitive (NS), Low (L), Medium (M), High (H) and Not relevant (NR) (Tillin & Tyler-Walters 2014) (Table 5).

The assessments also include semi-quantitative assessments of the quality of evidence, applicability of evidence and the degree of agreement between evidence sources. These were coded numerically and linked to the Plymouth Sound habitat data layer through a series of iterative joins, linking sensitivity information based on the most detailed habitat class information available (EUNIS levels 5 and 6), up to EUNIS level 3. At the higher EUNIS levels (3 and 4), MarESA assessments were aggregated, taking advantage of EUNIS’ hierarchical structure and following a precautionary approach to assign the most sensitive score of all ‘children’ classes from existing MarESA assessments to their ‘parent’ class.

This habitat-ES-sensitivity data layer was then intersected with data on fishing intensity. The fishing data used was an amalgamated product combining spatial information on smaller fishing vessels, obtained through the participatory mapping exercise FisherMap (des Clers *et al.* 2008), with aggregated VMS data for vessels over 15m (Enever *et al.* 2017). Enever *et al.* (2017) classified their dataset into low, medium or high exposure according to relative levels of fishing effort throughout English waters, based on quartiles of vessel counts per square nautical mile. These exposure levels were coded and combined spatially with the sensitivity information. Combinations of sensitivity and exposure levels were then used to indicate the likely impacts to benthic habitats, and their likely relative condition as a result (LRC). Figure 3 demonstrates the spatial representation of LRC across the study area. Table 6 provides the calculations for the area of the LRC of each habitat.

Figure 3: Likely Relative Condition (LRC) due to impacts from abrasion, as inferred from the sensitivity pressure approach. Map made in ArcGIS Pro by the University of Plymouth. Habitat data obtained from UKSeaMap: JNCC (Open Government License, https://hub.jncc.gov.uk/assets/202874e5-0446-4ba7-8323-24462077561e), EMODnet Seabed Habitats Initiative (emodnet-seabedhabitats.eu), the University of Plymouth and the Ocean Conservation Trust. Fishing data obtained from des Clers et al. (2008) and Enever et al. (2017).

Table 4: The Marine Evidence-based Sensitivity Assessment (MarESA) database (Tyler-Walters et al. 2018) for Zostera marine and noltei.

| **Habitat** | **Pressure** | **Sensitivity from MARESA or Sensitivity from literature** | **Resistance from MARESA (or impact on extent or health of habitat from other study)** | **Intolerance from MARESA (or impact on extent or health of habitat from other study)** | **Resilience / Recoverability from MARESA (or recovery time from other study)** |
| --- | --- | --- | --- | --- | --- |
| **Seagrass (Dwarf eelgrass) Zostera (Zosterella) noltei beds in littoral muddy sand** | Marine heatwaves (high) | High | Low | Not given | Very Low |
|  | Marine heatwaves (middle) | Medium | Medium | Not given | Very Low |
|  | Sea level rise (Extreme) | High | Low | Not given | Very Low |
|  | Sea level rise (high) | Medium | Medium | Not given | Very Low |
|  | Sea level rise (middle) | Medium | Medium | Not given | Very Low |
|  | Salinity increase (local) | Medium | Medium | Not given | Medium |
|  | Water flow (tidal current) changes (local) | Medium | Medium | Not given | Medium |
|  | Emergence regime changes | Medium | Medium | Not given | Medium |
|  | Wave exposure changes (local) | Medium | Medium | Not given | Medium |
|  | Nutrient enrichment | Medium | Medium | Not given | Medium |
|  | Organic enrichment | Medium | Medium | Not given | Medium |
|  | Physical loss (to land or freshwater habitat) | High | None | Not given | Very Low |
|  | Physical change (to another seabed type) | High | None | Not given | Very Low |
|  | Physical change (to another sediment type) | High | Low | Not given | Very Low |
|  | Habitat structure changes - removal of substratum (extraction) | High | None | Not given | Very Low |
|  | Abrasion/disturbance of the surface of the substratum or seabed | Medium | Low | Not given | Medium |
|  | Penetration or disturbance of the substratum subsurface | High | None | Not given | Low |
|  | Changes in suspended solids (water clarity) | High | Low | Not given | Low |
|  | Smothering and siltation rate changes (light) | Medium | Medium | Not given | Medium |
|  | Smothering and siltation rate changes (heavy) | High | None | Not given | Very Low |
|  | Introduction of light or shading | Medium | Low | Not given | Medium |
|  | Introduction or spread of invasive non-indigenous species | High | Low | Not given | Low |
|  | Introduction of microbial pathogens | Medium | Medium | Not given | Medium |
|  | Removal of non-target species | High | None | Not given | Low |
| **Seagrass (Common eelgrass) Zostera (Zostera) marina beds on lower shore or infralittoral clean or muddy sand** | Global warming (extreme) | Medium | Medium | Not given | Very Low |
|  | Global warming (high) | Medium | Medium | Not given | Very Low |
|  | Global warming (middle) | Medium | Medium | Not given | Very Low |
|  | Marine heatwaves (high) | High | Low | Not given | Low |
|  | Marine heatwaves (middle) | Medium | Medium | Not given | Medium |
|  | Sea level rise (extreme) | High | Low | Not given | Very Low |
|  | Sea level rise (high) | Medium | Medium | Not given | Very Low |
|  | Sea level rise (middle) | Medium | Medium | Not given | Very Low |
|  | Temperature increase (local) | Medium | Medium | Not given | Medium |
|  | Salinity increase (local) | Medium | Low | Not given | Medium |
|  | Salinity decrease (local) | Medium | Medium | Not given | Medium |
|  | Water flow (tidal current) changes (local) | Medium | Medium | Not given | Medium |
|  | Emergence regime changes | Medium | Low | Not given | Medium |
|  | Wave exposure changes (local) | Medium | Medium | Not given | Medium |
|  | Nutrient enrichment | Medium | Medium | Not given | Medium |
|  | Organic enrichment | Medium | Medium | Not given | Medium |
|  | Physical loss (to land or freshwater habitat) | High | None | Not given | Very Low |
|  | Physical change (to another seabed type) | High | None | Not given | Very Low |
|  | Physical change (to another sediment type) | High | Low | Not given | Very Low |
|  | Habitat structure changes - removal of substratum (extraction) | High | None | Not given | Very Low |
|  | Abrasion/disturbance of the surface of the substratum or seabed | Medium | Low | Not given | Medium |
|  | Penetration or disturbance of the substratum subsurface | High | None | Not given | Low |
|  | Changes in suspended solids (water clarity) | High | Low | Not given | Low |
|  | Smothering and siltation rate changes (light) | Medium | Low | Not given | Medium |
|  | Smothering and siltation rate changes (heavy) | High | None | Not given | Very Low |
|  | Introduction of light or shading | High | Low | Not given | Low |
|  | Introduction or spread of invasive non-indigenous species | High | Low | Not given | Low |
|  | Introduction of microbial pathogens | High | Low | Not given | Low |
|  | Removal of target species | Medium | None | Not given | Medium |
|  | Removal of non-target species | High | None | Not given | Low |

Table 5: The assessments assign scores for habitat sensitivity as a combination of resistance and resilience to particular pressures. The scores allocated are: Not Sensitive (NS), Low (L), Medium (M), High (H) and Not relevant (NR) (Tillin & Tyler-Walters 2014)

| Sensitivity | Exposure | | | |  | Sensitivity | Exposure | | | |
| --- | --- | --- | --- | --- | --- | --- | --- | --- | --- | --- |
|  | None | Low | Moderate | High |  |  | None | Low | Moderate | High |
| NS | None | None | None | None |  | NS | Good | Good | Good | Good |
| L | None | Low | Low | Moderate | ⇒ | L | Good |  |  | 🡫 |
| M | None | Low | Moderate | High |  | M | Good |  |  | 🡫 |
| H | None | Moderate | High | Very High |  | H | Good | 🡪 | 🡪 |  |

Table 6: Extents of Intertidal and Subtidal seagrass habitat assets, their extents within MPAs and the extent of the asset with a likely relative condition of less than or equal to moderate.

| Broad Habitat | Detail (with Eunis code) | Extent (km^2^) | % within MPA | Extent of site in LRC ≤ moderate (km^2^) |  |
| --- | --- | --- | --- | --- | --- |
| ***Marine inlets and transitional waters*** | | | |  |  |
| Intertidal sediments | Mainland Atlantic *Zostera noltei* or *Zostera marina* meadows (A2.611) | 0.261 | 0.92 % | 0 |  |
|  | *Zostera noltei* beds in littoral muddy sand (A2.6111) | 0.401 | 99.74 % | 0 |  |
| ***Sublittoral habitats*** |  |  |  |  |  |
| Subtidal sediment | Sublittoral seagrass beds (A5.53) | 0.346 | 99.73 % | 0.17 |  |
|  | *Zostera marina* beds on lower shore or infralittoral clean or muddy sand (A5.5331) | 0.169 | 99.99 % | 0.06 | |
| Totals | All Seagrass habitat | 0.963 | 77.86 % | 0.23 |  |

# The Condition of Seagrass Habitat at the individual Seagrass Bed level

To infer the condition of individual seagrass beds within the study area, data on individual seagrass beds was required. In the latest condition assessments of subtidal seagrass beds in Plymouth, some direct measurement of seagrass condition was reported for individual beds. Data from the most recent assessment is described below (Table 7, 8 and 9). There was no condition data on individual intertidal seagrass beds for the study area.

Key references:

Bunker F, Green B. 2019. *Seagrass condition monitoring in Plymouth Sound and Estuaries SAC 2018*.

Curtis LA. 2018. *Plymouth Sound and Estuaries SAC Littoral Habitats Condition Monitoring 2017: Report Number: ER17-348.* Ecospan Environmental Ltd.

Table 7: Extent and percentage change for seagrass habitat assets from the most recent survey of subtidal seagrass in the study area (Bunker & Green, 2019). 2018 Surveys from Tomb Rock (Yealm) and Red Cove North (Yealm) were different to the area surveyed in 2012 and hence couldn’t be compared (Bunker & Green, 2019). Jennycliff North is not included as seagrass was only recorded from a single DDV drop (Bunker & Green, 2019). Red cells show a decrease in area and Green cells show an increase in area.

|  |  | **Area Km^2^** | | **% Change** |
| --- | --- | --- | --- | --- |
|  |  | 2012 | 2018 |  |
| **Subtidal Seagrass** | Firestone Bay | 0.0076 | 0.0031 | -59 |
|  | Drake's Island | 0.0442 | 0.0407 | -8 |
|  | Jennycliff North |  |  |  |
|  | Jennycliff South | 0.0144 | 0.0063 | -56 |
|  | Cawsand Bay | 0.1197 | 0.1862 | 56 |
|  | Cellar’s Cove (Yealm) | 0.057 | 0.0473 | -17 |
|  | Red Cove North (Yealm) | 0.0262 |  |  |
|  | Red Cove South (Yealm) | 0.0114 | 0.0116 | 1 |
|  | Tomb Rock (Yealm) | 0.0663 |  |  |

Table 8: Subtidal seagrass average percentage cover changes from Bunker and Green (2019) DDV surveys. Surveys from Tomb Rock (Yealm) and Red Cove North (Yealm) were different to the area surveyed in 2012 and hence couldn’t be compared (Bunker & Green, 2019). In both 2012 and 2018, cover data from Jennycliff North, is based on a single DDV drop (data record) and so may not be an accurate representation of the seagrass bed (Bunker & Green, 2019). Red cells show a decrease in cover and Green cells show an increase in cover.

|  | **Average % Cover DDV** | |
| --- | --- | --- |
|  | 2012 | 2018 |
| Firestone Bay | 21 | 17 |
| Drake's Island | 72 | 66 |
| Jennycliff North | 70 | 6 |
| Jennycliff South | 21 | 14 |
| Cawsand Bay | 30 | 59 |
| Cellar's Cove (Yealm) | 74 | 69 |
| Red Cove South (Yealm) | 80 | 55 |
| Red Cove North (Yealm) | 77 | X |
| Tomb Rock (Yealm) | 19 | X |

Table 9: Subtidal seagrass direct condition indicator changes between 2012 and 2018. Gathered from in situ dive surveys (Bunker & Green, 2019). Red cells show where there has been a decrease in seagrass condition, and green where there has been an increase in seagrass condition.

|  | **Av Shoot Density m^2^** | | **Av Plant Length (cm)** | | **% Infected Leaves** | |
| --- | --- | --- | --- | --- | --- | --- |
|  | **2012** | **2018** | **2012** | **2018** | **2012** | **2018** |
| Drake's Island | 97 | 64 | 54 | 80 | 55 | 53 |
| Cawsand Bay | 34 | 86 | 34 | 54 | 42 | 41 |
| Cellar's Cove (Yealm) | 122 | 112 | 63 | 52 | 50 | 53 |
| Red Cove South (Yealm) | 134 | 119 | 50 | 56 | 56 | 29 |

**Water Quality Data**

Water quality and clarity contribute to the health of seagrass. Common issues for seagrass relating to water quality and clarity are nutrient loading (the increased concentration of nutrients) which can increase the growth of competitive opportunistic macroalgae and epiphytes, reducing the amount of light available to seagrass for photosynthesis (Burkholder, Tomasko & Touchette, 2007). This can consequently impact metabolism, growth, and reproduction. Similarly, high turbidity and poor water clarity can mean increased light attenuation and less photosynthetic active radiation (PAR) (Erftemeijer & Robin Lewis, 2006).

Water body status data, in reference to WFD targets, provides information on local water quality. Data on water body status was accessed. Water bodies are required to have all status categories classified as ‘good’ or ‘high’ to meet WFD requirements (European Commission, 2014). Surface Water Status (often referred to as Overall Status) is the water body level classification derived from combining both Chemical Status and Ecological Status (EA, 2015). There are four water bodies within the PSE site, all of which overlap with seagrass habitat assets (Table 10; Figure 4).

In 2019, the most recent year available for water body status data, all four water bodies within the PSE site failed to meet WFD standards with a status of ‘moderate’ (Tables 11, 12, 13, and 14). For all four water bodies, Reasons for Not Achieving ‘Good’ status (RNAG) included levels of mercury and polybrominated diphenyl ethers (PBDE) that failed to meet required standards. Failed chemical standards for water bodies in the PSE area could be due to historic pollution associated with the mining industry (Ashley, Rees & Mullier, 2021). It is assumed that Organotin contamination in the Tamar, originated from marinas and the Devonport Dockyard. Though this source of contamination likely stopped in the 1980s it is suspected that sediments now also contribute (Langston *et al.*, 2003). Contaminants that are locked into sediments can be remobilised and released back into the water column through activities like dredging (Ashley, Rees & Mullier, 2021).

In Plymouth Sound water body, Dissolved Inorganic Nitrogen (DIN) has had a status of ‘moderate’ since 2013 contributing to the overall ‘moderate’ status for the water body. The Environment Agency (EA) lists sewage discharge and agricultural land management as the likely cause of moderate DIN status (EA, 2021). The risk of eutrophication (from high nutrient loads) across all four water bodies is expected to be low due to ‘good – high’ status of phytoplankton and macroalgae since 2013. In NE’s condition assessments, these biological indicators of eutrophication (phytoplankton blooms and opportunistic macroalgae) are used to assess the risk of eutrophication to seagrass (EA, 2021; NE, 2021).

Figure 5 shows the contribution of combined sewage overflows (CSOs) to the study site. The results represent the ‘counted spills’ using the 12-24 hour counting method (EA, 2018). Overall there were 393 spills in 2021 into Plymouth Sound, which totaled a duration of 1510 hours (Total Duration (hrs) all spills prior to processing through 12-24h count method) (DEFRA, 2022).

CSOs create pathways for potential pathogens to enter the waters of the Sound, and these risks can be more pronounced where the water aggregates. Sewage poses an obvious risk to human health (see <https://plymouthmarineforecasts.org/Tools/Pollution_risk_tool_en>) and amenity value. There is a growing body of evidence from international studies that seagrass species function to remove pathogens, therefore contribute to improved sanitation with expected benefits for human health risk (Unsworth *et al.*, 2022).

Table 10: Table describing the overlap in water body area with intertidal and subtidal seagrass habitat assets

| Water Body Name | Plymouth Sound | Plymouth Coast | Plymouth Tamar | Yealm Estuary |
| --- | --- | --- | --- | --- |
| Water Body ID | GB650806230000 | GB620806110003 | GB520804714300 | GB520804706200 |
| Intertidal Seagrass |  |  |  |  |
| Subtidal Seagrass |  |  |  |  |

Figure 4: Study site water bodies and overlap with seagrass habitat assets. Map made in ArcGIS Pro by the University of Plymouth. Habitat data obtained from UKSeaMap: JNCC (Open Government License, https://hub.jncc.gov.uk/assets/202874e5-0446-4ba7-8323-24462077561e), EMODnet Seabed Habitats Initiative (emodnet-seabedhabitats.eu), the University of Plymouth and the Ocean Conservation Trust. Water body data obtained from the Environment Agency (Open Government Liscence, https://www.gov.uk/guidance/water-framework-directive-assessment-estuarine-and-coastal-waters).

Figure 5: Combined Sewage Overflow (CSO) distribution and outflow intensity for the study site area. Map made in ArcGIS Pro by the University of Plymouth. CSO data obtained from the Environment Agency (Open Government Liscence, https://environment.data.gov.uk/dataset/21e15f12-0df8-4bfc-b763-45226c16a8ac).

Table 11: Plymouth Sound water body against WFD targets.

| **Classification Item** | **2013** | **2014** | **2015** | **2016** | **2019** |
| --- | --- | --- | --- | --- | --- |
| **Overall** | **Moderate** | **Moderate** | **Moderate** | **Moderate** | **Moderate** |
| **Ecological** | **Moderate** | **Moderate** | **Moderate** | **Moderate** | **Moderate** |
| **Biological quality elements** | **Good** | **Good** | **Good** | **Good** | **Good** |
| Invertebrates | **Good** | **Good** | **Good** | **Good** | **Good** |
| Imposex | **Good** | **Good** | **Good** | **Good** | **Good** |
| Infaunal Quality Index | **Good** | **Good** | **Good** | **Good** | **Good** |
| Macroalgae | **High** | **High** | **High** | **High** | **High** |
| Rocky Shore Macroalgae | **High** |  | **High** | **High** | **High** |
| Phytoplankton | **High** | **High** | **High** | **High** | **Good** |
| **Physico-chemical quality elements** | **Moderate** | **Moderate** | **Moderate** | **Moderate** | **Moderate** |
| Dissolved Inorganic Nitrogen | **Moderate** | **Moderate** | **Moderate** | **Moderate** | **Moderate** |
| Dissolved oxygen | **High** | **High** | **High** | **High** | **High** |
| **Hydromorphological Supporting Elements** | **Supports good** | **Supports good** | **Supports good** | **Supports good** | **Supports good** |
| Morphology | **Supports good** | **Supports good** | **Supports good** | **Supports good** | **Supports good** |
| **Specific pollutants** | **High** | **High** | **High** | **High** | **High** |
| Arsenic | **High** | **High** | **High** | **High** | **High** |
| Chromium (VI) |  |  |  |  | **High** |
| Copper | **High** | **High** | **High** | **High** | **High** |
| Iron | **High** | **High** | **High** | **High** | **High** |
| Triclosan | **High** | **High** |  |  |  |
| Un-ionised ammonia |  | **High** | **High** | **High** | **High** |
| Zinc | **High** | **High** | **High** | **High** | **High** |
| **Other Substances** |  |  |  |  |  |
| 1-1-1-trichloroethane | **High** |  |  |  |  |
| **Chemical** | **Fail** | **Fail** | **Good** | **Good** | **Fail** |
| **Priority hazardous substances** | **Fail** | **Fail** | **Good** | **Good** | **Fail** |
| Benzo(a)pyrene |  |  |  |  | **Good** |
| Benzo(b)fluoranthene |  |  |  |  | **Good** |
| Benzo(g-h-i)perylene |  |  |  |  | **Fail** |
| Benzo(k)fluoranthene |  |  |  |  | **Good** |
| Cadmium and Its Compounds | **Good** | **Good** | **Good** | **Good** | **Good** |
| Di(2-ethylhexyl)phthalate (Priority hazardous) | **Good** | **Good** |  |  |  |
| Dioxins and dioxin-like compounds |  |  |  |  | **Good** |
| Hexabromocyclododecane (HBCDD) |  |  |  |  | **Good** |
| Hexachlorobenzene |  |  |  |  | **Good** |
| Hexachlorobutadiene |  |  |  |  | **Good** |
| Mercury and Its Compounds |  |  | **Good** | **Good** | **Fail** |
| Nonylphenol | **Good** | **Good** | **Good** | **Good** | **Good** |
| Perfluorooctane sulphonate (PFOS) |  |  |  |  | **Good** |
| Polybrominated diphenyl ethers (PBDE) |  |  |  |  | **Fail** |
| Tributyltin Compounds | **Fail** | **Fail** |  |  | **Good** |
| **Priority substances** | **Good** | **Good** | **Good** | **Good** | **Good** |
| Fluoranthene |  |  |  |  | **Good** |
| Lead and Its Compounds | **Good** | **Good** | **Good** | **Good** | **Good** |
| Nickel and Its Compounds | **Good** | **Good** | **Good** | **Good** | **Good** |
| Octylphenol |  |  |  |  | **Good** |
| Trichloromethane | **Good** | **Good** | **Good** | **Good** | **Good** |
| **Other Pollutants** | **Does not require assessment** | **Does not require assessment** | **Does not require assessment** | **Does not require assessment** | **Does not require assessment** |

Table 12: Plymouth Coast water body against WFD targets.

| **Classification Item** | **2013** | **2014** | **2015** | **2016** | **2019** |
| --- | --- | --- | --- | --- | --- |
| **Overall** | **Moderate** | **Moderate** | **Good** | **Good** | **Moderate** |
| **Ecological** | **Good** | **Good** | **Good** | **Good** | **Good** |
| **Biological quality elements** | **Good** | **Good** | **Good** | **Good** | **Good** |
| Invertebrates | **Good** | **Good** | **Good** | **Good** | **Good** |
| Infaunal Quality Index | **Good** | **Good** | **Good** | **Good** | **Good** |
| Phytoplankton | **High** | **High** | **High** | **High** | **High** |
| **Physico-chemical quality elements** | **Good** | **High** | **High** | **High** | **High** |
| Dissolved Inorganic Nitrogen | **Good** | **High** | **High** | **High** | **High** |
| Dissolved oxygen | **High** | **High** | **High** | **High** | **High** |
| **Hydromorphological Supporting Elements** | **Supports good** | **Supports good** | **Supports good** | **Supports good** | **Supports good** |
| Morphology | **Supports good** | **Supports good** | **Supports good** | **Supports good** | **Supports good** |
| **Chemical** | **Fail** | **Fail** | **Good** | **Good** | **Fail** |
| **Priority hazardous substances** | **Fail** | **Fail** | **Good** | **Good** | **Fail** |
| Benzo(a)pyrene | **Good** | **Good** |  |  | **Good** |
| Dioxins and dioxin-like compounds |  |  |  |  | **Good** |
| Heptachlor and cis-Heptachlor epoxide |  |  |  |  | **Good** |
| Hexabromocyclododecane (HBCDD) |  |  |  |  | **Good** |
| Hexachlorobenzene | **Good** | **Good** | **Good** | **Good** | **Good** |
| Hexachlorobutadiene | **Good** | **Good** | **Good** | **Good** | **Good** |
| Mercury and Its Compounds | **Fail** | **Fail** |  |  | **Fail** |
| Perfluorooctane sulphonate (PFOS) |  |  |  |  | **Good** |
| Polybrominated diphenyl ethers (PBDE) |  |  |  |  | **Fail** |
| **Priority substances** | **Good** | **Good** | **Does not require assessment** | **Does not require assessment** | **Good** |
| Fluoranthene | **Good** | **Good** |  |  | **Good** |
| **Other Pollutants** | **Does not require assessment** | **Does not require assessment** | **Does not require assessment** | **Does not require assessment** | **Does not require assessment** |

Table 13: Plymouth Tamar water body against WFD targets.

| **Classification Item** | **2013** | **2014** | **2015** | **2016** | **2019** |
| --- | --- | --- | --- | --- | --- |
| **Overall** | **Moderate** | **Moderate** | **Moderate** | **Moderate** | **Moderate** |
| **Ecological** | **Good** | **Good** | **Moderate** | **Moderate** | **Moderate** |
| **Biological quality elements** | **Good** | **Good** | **Good** | **Good** | **Good** |
| Angiosperms |  |  |  |  | **High** |
| Seagrass |  |  |  |  | **High** |
| Invertebrates | **Good** | **Good** | **Good** | **Good** | **Good** |
| Infaunal Quality Index | **Good** | **Good** | **Good** | **Good** | **Good** |
| Macroalgae | **Good** | **Good** | **Good** | **Good** | **Good** |
| Opportunistic Macroalgae | **Good** | **Good** | **Good** | **Good** | **Good** |
| **Physico-chemical quality elements** | **High** | **High** | **High** | **High** | **High** |
| Dissolved oxygen | **High** | **High** | **High** | **High** | **High** |
| **Hydromorphological Supporting Elements** | **Supports good** | **Supports good** | **Supports good** | **Supports good** | **Supports good** |
| Hydrological Regime | **Supports good** | **Supports good** | **Supports good** | **Supports good** | **Supports good** |
| **Supporting elements (Surface Water)** | **Good** | **Good** | **Moderate** | **Moderate** | **Moderate** |
| Mitigation Measures Assessment | **Good** | **Good** | **Moderate or less** | **Moderate or less** | **Moderate or less** |
| **Specific pollutants** | **High** | **High** | **High** | **High** | **High** |
| Arsenic | **High** | **High** | **High** | **High** | **High** |
| Copper | **High** | **High** | **High** | **High** | **High** |
| Iron |  | **High** | **High** | **High** | **High** |
| Zinc | **High** | **High** | **High** | **High** | **High** |
| **Chemical** | **Fail** | **Fail** | **Good** | **Good** | **Fail** |
| **Priority hazardous substances** | **Fail** | **Fail** | **Good** | **Good** | **Fail** |
| Benzo(a)pyrene | **Good** | **Good** |  |  | **Good** |
| Cadmium and Its Compounds | **Good** | **Good** | **Good** | **Good** | **Good** |
| Dioxins and dioxin-like compounds |  |  |  |  | **Good** |
| Hexabromocyclododecane (HBCDD) |  |  |  |  | **Good** |
| Hexachlorobenzene |  |  |  |  | **Good** |
| Hexachlorobutadiene |  |  |  |  | **Good** |
| Mercury and Its Compounds | **Good** | **Good** |  |  | **Fail** |
| Perfluorooctane sulphonate (PFOS) |  |  |  |  | **Good** |
| Polybrominated diphenyl ethers (PBDE) |  |  |  |  | **Fail** |
| Tributyltin Compounds | **Fail** | **Fail** |  |  | **Fail** |
| **Priority substances** | **Good** | **Good** | **Good** | **Good** | **Good** |
| Fluoranthene | **Good** | **Good** |  |  | **Good** |
| Lead and Its Compounds | **Good** | **Good** | **Good** | **Good** | **Good** |
| Nickel and Its Compounds | **Good** | **Good** | **Good** | **Good** | **Good** |
| **Other Pollutants** | **Does not require assessment** | **Does not require assessment** | **Does not require assessment** | **Does not require assessment** | **Does not require assessment** |

Table 14: Yealm water body against WFD targets.

| **Classification Item** | **2013** | **2014** | **2015** | **2016** | **2019** |
| --- | --- | --- | --- | --- | --- |
| **Overall** | **Moderate** | **Moderate** | **Good** | **Good** | **Moderate** |
| **Ecological** | **Good** | **Good** | **Good** | **Good** | **Good** |
| **Biological quality elements** | **Good** | **Good** | **Good** | **Good** | **Good** |
| Macroalgae | **Good** | **Good** | **Good** | **Good** | **Good** |
| Opportunistic Macroalgae | **Good** | **Good** | **Good** | **Good** | **Good** |
| **Physico-chemical quality elements** | **High** | **High** | **High** | **High** | **High** |
| Dissolved oxygen | **High** | **High** | **High** | **High** | **High** |
| **Hydromorphological Supporting Elements** | **Supports good** | **Supports good** | **Supports good** | **Supports good** | **Supports good** |
| Hydrological Regime | **Supports good** | **Supports good** | **Supports good** | **Supports good** | **Supports good** |
| Morphology | **High** | **High** | **High** | **High** | **High** |
| **Specific pollutants** | **High** | **High** | **High** | **High** | **High** |
| Arsenic | **High** | **High** | **High** | **High** | **High** |
| Copper | **High** | **High** | **High** | **High** | **High** |
| Zinc | **High** | **High** | **High** | **High** | **High** |
| **Chemical** | **Fail** | **Fail** | **Good** | **Good** | **Fail** |
| **Priority hazardous substances** | **Fail** | **Fail** | **Good** | **Good** | **Fail** |
| Benzo(a)pyrene |  |  |  |  | **Good** |
| Cadmium and Its Compounds | **Good** | **Good** | **Good** | **Good** | **Good** |
| Dioxins and dioxin-like compounds |  |  |  |  | **Good** |
| Hexabromocyclododecane (HBCDD) |  |  |  |  | **Good** |
| Hexachlorobenzene |  |  |  |  | **Good** |
| Hexachlorobutadiene |  |  |  |  | **Good** |
| Mercury and Its Compounds |  |  |  |  | **Fail** |
| Perfluorooctane sulphonate (PFOS) |  |  |  |  | **Good** |
| Polybrominated diphenyl ethers (PBDE) |  |  |  |  | **Fail** |
| Tributyltin Compounds | **Fail** | **Fail** |  |  | **Good** |
| **Priority substances** | **Good** | **Good** | **Good** | **Good** | **Good** |
| Fluoranthene |  |  |  |  | **Good** |
| Lead and Its Compounds | **Good** | **Good** | **Good** | **Good** | **Good** |
| Nickel and Its Compounds | **Good** | **Good** | **Good** | **Good** | **Good** |
| **Other Pollutants** | **Does not require assessment** | **Does not require assessment** | **Does not require assessment** | **Does not require assessment** | **Does not require assessment** |

# Habitat Suitability Data

Data from Table 15 was used to generate the habitat suitability maps for *Zostera marina* and *noltei.*

Table 15: Habitat Suitability Data from the Marlin online database.

| **Habitat** | **Broad habitat association** | **Eunis level 1 habitat association** | **Other associated habitat detail** | **Depth range** | **Salinity range (or zone)** | **Exposure to wave action tolerated** | **Turbidity / water clarity range tolerated** | **Limiting nutrients** | **Other limiting environmental factors identified** |
| --- | --- | --- | --- | --- | --- | --- | --- | --- | --- |
|  |  |  |  |  |  |  |  |  |  |
| **Seagrass (Dwarf eelgrass) Zostera (Zosterella) noltei beds in littoral muddy sand** | Enclosed coast, embayment | Littoral sediment | Muddy sand, Sandy mud | Upper shore, Mid shore | Full (30-40 psu), Variable (18-40 psu) | Extremely sheltered, Sheltered, Very sheltered |  | Nitrogen (nitrates), Phosphorus (phosphates) | Tidal Strength Preference: Moderately Strong 1 to 3 knots (0.5-1.5 m/sec.), Very Weak (negligible), Weak < 1 knot (<0.5 m/sec.) (not same link - <https://www.marlin.ac.uk/species/detail/1409>) |
| **Seagrass (Common eelgrass) Zostera (Zostera) marina beds on lower shore or infralittoral clean or muddy sand** | Enclosed coast / Embayment | Sublittoral Sediment | Mud, Mud and sandy mud, Muddy sand, Sand, Sand and muddy sand | Lower shore, 0-5 m, 5-10 m | Full (30-40 psu), Variable (18-40 psu) | Extremely sheltered, Moderately exposed, Sheltered, Very sheltered |  | Nitrogen (nitrates), Phosphorous (phosphates) | Tidal Strength Preference: Moderately Strong 1 to 3 knots (0.5-1.5 m/sec.), Very Weak (negligible), Weak < 1 knot (<0.5 m/sec.) |

Table 16: Environmental predictor data sets for habitat suitability modelling.

| **Environmental Variable** | **Source** | **Units** | **Spatial Resolution** | **Version Date** |
| --- | --- | --- | --- | --- |
| Underlying habitat type | Composite habitat layer created from survey data provided by EMODnet seabed habitats and UKSeaMap 2018v2. | EUNIS habitat classification | Variable (composite data set) | Variable |
| Bathymetry (depth) | OceanWise Marine Themes Digital Elevation Model. Accessed via EDINA Marine Digimap Service, downloaded 2021-06-24. | Metres (m) | 1 arc-second | 2021 |
| Kinetic energy at the seabed due to waves | UKSeaMap 2018v2 | Newtons per Square Metre (N/m^2^) | 3arc-second | 2018 |

Key references:

Ashley M, Rees S, Mullier T. 2021. *Natural Capital Asset and Risk Register to Inform Marine Site Management Plans and Implementation of Plymouth National Marine Park. Part One: Introduction to Natural Capital Assets and Ecosystem Service Benefits within Plymouth Sound, Estuaries and Coastal Area. Report compiled by staff at the University of Plymouth.*

Burkholder JM, Tomasko DA, Touchette BW. 2007. Seagrasses and eutrophication. *Journal of Experimental Marine Biology and Ecology* 350:46–72. DOI: https://doi.org/10.1016/j.jembe.2007.06.024.

d’Avack EAS, Tyler-Walters H, Wilding CM, Garrard SL. 2022. Zostera (Zostera) marina beds on lower shore or infralittoral clean or muddy sand. In Tyler-Walters H. and Hiscock K. (eds) Marine Life Information Network: Biology and Sensitivity Key Information Reviews.

d’Avack EAS, Tyler-Walters H, Wilding CM, Garrard SL. 2022. Zostera (Zosterella) noltei beds in littoral muddy sand. In Tyler-Walters H. and Hiscock K. (eds) Marine Life Information Network: Biology and Sensitivity Key Information Reviews.

DEFRA. 2022. Event Duration Monitoring - Storm Overflows - Annual Returns.

Environment Agency. 2015.Rules for assessing Surface Water Body Status and Potential. In: DEFRA (ed.).

Environment Agency. 2021.WFD Classification Status Cycle 2. *Available at* *https://data.gov.uk/dataset/41cb73a1-91b7-4a36-80f4-b4c6e102651a/wfd-classification-status-cycle-2.*

Enever R, Lewin S, Reese A, Hooper T. 2017. Mapping fishing effort: Combining fishermen’s knowledge with satellite monitoring data in English waters. *FISHERIES RESEARCH* 189:67–76. DOI: 10.1016/j.fishres.2017.01.009.

Erftemeijer PLA, Robin Lewis RR. 2006. Environmental impacts of dredging on seagrasses: A review. *Marine Pollution Bulletin* 52:1553–1572. DOI: https://doi.org/10.1016/j.marpolbul.2006.09.006.

European Commission D-G for E. 2014. The EU Water Framework Directive. Publications Office.

Langston WJ, Chesman BS, Burt GR, Hawkins SJ, Readman JW, Worsfold P. 2003. Characterisation of the South West European Marine Sites: Plymouth Sound and Estuaries cSAC, SPA. Occasional Publication of the Marine Biological Association 9.

Natural England. 2021.Plymouth Sound and Estuaries SAC. Feature Condition. *Available at* *https://designatedsites.naturalengland.org.uk/Marine/MarineFeatureCondition.aspx?SiteCode=UK0013111&SiteName=plymouth&SiteNameDisplay=Plymouth+Sound+and+Estuaries+SAC&countyCode=&responsiblePerson=&SeaArea=&IFCAArea=*

Tillin H, Tyler-Walters H. 2014.Assessing the sensitivity of subtidal sedimentary habitats to pressures associated with marine activities – Phase 2 Report | JNCC Resource Hub. *Available at* *https://hub.jncc.gov.uk/assets/742cd48d-4349-4894-bbbf-d38b1c158a1c* (accessed July 3, 2024).

Unsworth RKF, Cullen-Unsworth LC, Jones BLH, Lilley RJ. 2022. The planetary role of seagrass conservation. *Science* 377:609–613. DOI: doi:10.1126/science.abq6923.
