# Supplementary material for: Using a natural capital risk register to support the funding of seagrass habitat enhancement in Plymouth Sound
Source: PeerJ. 2024 Oct 28;12:e17969. doi: 10.7717/peerj.17969 (PMC11526789; doi:10.7717/peerj.17969)
Supplement: Supplemental Information 2 [file peerj-12-17969-s002.docx]

**Supplementary Material 2:**

**Policy targets and justification for risk register scoring**

We set out here the policy targets (Table 1) and the assessment against these relating to the 120 asset benefit relationships, where there is a moderate to high link between the extent, condition or spatial configuration of the habitat assets and the flow of benefits (Table 3). The Extent, Condition or Spatial Configuration status and trend of the seagrass assets are assessed in relation to a defined policy target (Table 1).

Table 2 below explains how the following asset and risk register can be read, following Mace et al., (2015). Using the evidence from Supplementary Material 1 the status and trend in data were assessed. The R (Red), Amber (A), Green (G) score is based on assessment (led by GH and verified by the wider team and project Steering Group) of the asset status in relation to the policy target and the trend over time. Each RAG rating was assessed for the strength of evidence and agreement between data input sources on a scale of 1-4 for both status and trend. The overall confidence score is the sum of confidence scores for status and trend. Mace *et al*. (2015) presented total scores <4 as high confidence (low uncertainty), and scores of >5 as low confidence in the evidence, and so high uncertainty. In this study we have applied a precautionary approach and clarified this scoring with total scores of between 1 to 3 regarded as high confidence in the RAG rating (low uncertainty) and total scores of ≥4 regarded as low confidence in the RAG rating (high uncertainty).

Table 1: Summarised policy targets for seagrass habitat assets across national (UK) and international policies

| Seagrass Asset Status | Indicator | Policy | Target |
| --- | --- | --- | --- |
| Extent | Area of habitat (km^2^) | CBD Aichi 11; SDG 14 | 10% of habitat extent within MPA |
|  |  | Marine Strategy Framework Directive (MSFD) Descriptor 1 | Target for all soft substratum habitats (where extent of the habitat is less than 50% of the assessed region). **Inside MPAs:** extent is stable or increasing (>95% conservation objective 'maintain'. **Outside MPAs:** area of habitat lost + area of habitat below GES (in condition recover or impacted by unacceptable impact (*UoP interpretation* LRC 3 or below) ≤ 10% for entire assessed area.  Target for all soft substratum habitats (where extent of the habitat is above 50% of all assessed area). **Inside MPAs:** extent is stable or increasing (>95% conservation objective 'maintain'). **Outside MPAs:** area of habitat lost + area of habitat below GES (in condition recover or impacted by unacceptable impact *UoP interpretation* (LRC 3 or below) ≤ 15% for entire assessed area. |
|  |  | UK Marine Strategy | Habitat extent shows no loss of extent from previous assessment.  *UoP interpretation Qualitative threshold:* Area in LRC of moderate or below is less than 5% of total habitat extent.  *Quantitative threshold:* Area assessed in condition assessments to be in unfavourable condition is less than 5% of total habitat extent.  *UoP interpretation* Area with LRC of moderate or below is less than 5% of total habitat extent. |
| Condition | Area of each habitat within MPAs with conservation objective to be maintain or restore | MSFD Descriptor 6 | Pressure based target: extent affected by human disturbance should be minimised, and physical loss is minimised and where possible reversed (Defra, 2019): **Inside MPAs:** ≥95% of extent to be in favourable condition. i) presence and spatial distribution of biological communities’ representative of the feature are maintained. ii) presence and abundance of key structural and influential species are maintained (≥95% of extent to have conservation objective ‘maintain’). **Outside MPAs:** as for ‘extent’. |
|  | Area of each habitat outside MPAs with a modelled LRC of 3≥ |  |  |
|  | Area of adversely impacted habitat (km^2^) | UK Marine Strategy | *Listed habitats:* Area assessed in condition assessments to be in favourable condition with less than 5% unfavourable.  If proportion of extent assessed as unfavourable in previous assessment, then this proportion to have decreased.  UoP interpretation  Area in LRC of moderate or below is less than 5% of total habitat extent.  Area in LRC of moderate or below to have reduced since last assessment. |
| Condition | Area of adversely impacted habitat (km^2^) | Water Framework Directive | *WFD Ecological Quality Ratios for seagrass within WFD boundaries.*  Water body ecological, chemical and overall condition to be good or better.  DO and DIN levels to meet WFD targets in water body data. |
| Spatial Configuration | **Inside MPAs** and **Outside MPAs** were assessed as for ‘extent’. Unless specific conservation objectives contained | | |

Table 2: Template for Asset and Risk Register.

| Habitat / Species Asset | Benefit | Characteristic | Current Status | Target | Trend | RAG (A-C) |
| --- | --- | --- | --- | --- | --- | --- |
|  | **• Bold text = med to significant contribution.**  • Light text = low contribution | Characteristic of the asset being assessed: Extent, Condition or Spatial Configuration.  Condition sets out production functions, within underlying natural capital assets. Where available indicators were assessed that can be influenced and are important to provision of ES benefits. | What is the status of the relationship relative to a defined target? |  | What is the trend in the relationship? | RAG  (Overall RAG based on status and trend)  Total Uncertainty  (Summation of Uncertainty) |
|  |  |  | RAG rating for status | | RAG rating for trend |  |
|  |  |  | Uncertainty of status | | Uncertainty of trend |  |

|  |  | Agreement | |
| --- | --- | --- | --- |
|  |  | High | Low |
| Robustness | Significant evidence | 1 | 3 |
|  | limited evidence | 2 | 4 |

|  |  | Status | | |
| --- | --- | --- | --- | --- |
|  |  | Above, at or just below target | Below target | Substantially below target |
| Trend in Status | Positive or not discernible | A | B | B |
|  | Negative | B | B* | C |
|  | Strongly Negative | C | C | C |

|  |  | Status | | |
| --- | --- | --- | --- | --- |
|  |  | Above, at or just below target | Below target | Substantially below target |
| Trend in Status | Positive or not discernible | Low | Medium | Medium |
|  | Negative | Medium | Medium* | High |
|  | Strongly Negative | High | High | High |

|  | High confidence | Low confidence |
| --- | --- | --- |
| Low risk | A | A |
| High risk (or risk unknown) | B-B* | B-B* |
| Very high risk | C | C |

Table 3: Seagrass Asset and Risk Register

| **Broad Habitat type** | **Habitat / Species Asset** | **Benefit** | **Characteristic** | **Current Status** | **Target** | **Trend** | **RAG** |
| --- | --- | --- | --- | --- | --- | --- | --- |
| Coastal Margin | Intertidal Seagrass | **• Food (Wild Food - fish and shellfish).**  **• Healthy climate (carbon sequestration). • Sea defence. (natural hazard regulation). • Tourism/nature watching. • Clean water and sediments.** | Quantity/Extent | **Composite mapping**: Intertidal seagrass bed extent is at least 0.66 km^2^. 0.44 km^2^ of this area exists inside an MPA (composite habitat mapping).  **NE Package:** No data on change in extent of this feature. Conservation objective to maintain extent of habitat has been met. **(Low Confidence)** | **MSFD:** target for all soft substratum habitats (where extent of the habitat is less than 50% of the assessed region). Inside MPAs: extent is stable or increasing (>95% conservation objective 'maintain'. Outside MPAs: area of habitat lost + area of habitat below GES (in condition recover or impacted by unacceptable impact (UoP interpretation LRC 3 or below) ≤ 10% for entire assessed area.  **UK Marine** Strategy**:** Habitat extent shows no loss of extent from previous assessment.  *UoP interpretation Qualitative threshold:* Area in LRC of moderate or below is less than 5% of total habitat extent.  *Quantitative threshold:* Area assessed in condition assessments to be in unfavourable condition is less than 5% of total habitat extent.  *UoP interpretation* Area in LRC of moderate or below is less than 5% of total habitat extent. | No data on Trend. | **B (8)** |
|  |  |  |  | B (low confidence) – last assessment date 2018 | | B |  |
|  |  |  |  | (4) | | (4) |  |
|  | Intertidal Seagrass | **• Food (Wild Food - fish and shellfish).**  **• Healthy climate (carbon sequestration). • Sea defence. (natural hazard regulation). • Tourism/nature watching. • Clean water and sediments.** | Quality/Condition | **NE Package**: Within the Plymouth Sound and Estuaries SAC, Intertidal Seagrass has a feature condition of “Unfavourable Unknown”.  Two secondary attributes fail, both are proxies. 1) Sediment Contaminants and 2) ephemeral macroalgae (Opportunistic macroalgae covering and competing with the seagrass).  **LRC:** all intertidal seagrass mapped has a LRC score greater than moderate.  **Water Quality:** Since 2013, Plymouth Tamar water body has failed to meet Water Framework Directive (WFD) standards, achieving an overall water body status of ‘moderate’. In 2019, this was due to chemical hazardous substances failing against WFD standards. Seagrass is listed as a biological quality element that contributes to the ecological and overall status of the water body. In 2019, Seagrass was “high”suggesting intertidal seagrass was in good condition. This rating is based of an EQR. “The intertidal seagrass tool is an index based on the average of three criteria: seagrass bed extent loss, annual/five-yearly average shoot density loss, and species loss as a proportion of a historical reference. These measurements are combined to derive an Ecological Quality Ratio” (CEFAS) | **MSFD**: Pressure based target: extent affected by human disturbance should be minimised, and physical loss is minimised and where possible reversed (Defra, 2019): Inside MPAs: ≥95% of extent to be in favourable condition. i) presence and spatial distribution of biological communities’ representative of the feature are maintained. ii) presence and abundance of key structural and influential species are maintained (≥95% of extent to have conservation objective ‘maintain’). Outside MPAs: as for ‘extent’.  **UK Marine Strategy**: Listed habitats: Area assessed in condition assessments to be in favourable condition with less than 5% unfavourable. If proportion of extent assessed as unfavourable in previous assessment, then this proportion to have decreased.  UoP interpretation  Area in LRC of moderate or below is less than 5% of total habitat extent.  Area in LRC of moderate or below to have reduced since last assessment.  **Water Framework Directive:** WFD Ecological Quality Ratios for seagrass within WFD boundaries.  Water body ecological, chemical and overall condition to be good or better.  DO and DIN levels to meet WFD targets in water body data. | Unfavourable unknown condition. No data for comparison. Majority of conservation objectives to maintain. | **B (8)** |
|  |  |  |  | B – (low confidence) – last assessment date 2018 | | B |  |
|  |  |  |  | (4) | | (4) |  |
|  | Intertidal Seagrass | **• Food (Wild Food - fish and shellfish).**  **• Healthy climate (carbon sequestration). • Sea defence. (natural hazard regulation). • Tourism/nature watching. • Clean water and sediments.** | Spatial configuration | NE Package:  Conservation objectives 1) Maintain the total extent and spatial distribution of seagrass beds, 2) Maintain the area of habitat which is likely to support the subfeature 3) Maintain the presence and spatial distribution of intertidal seagrass bed communities; were all ‘met’ but with low confidence | As for extent. | Unknown | **B (8)** |
|  |  |  |  | B | | B |  |
|  |  |  |  | (4) | | (4) |  |

| **Broad Habitat type** | **Habitat / Species Asset** | **Benefit** | **Characteristic** | **Current Status** | **Target** | **Trend** | **RAG** |
| --- | --- | --- | --- | --- | --- | --- | --- |
| Coastal Margin | Subtidal Seagrass | **• Food (Wild Food - fish and shellfish).**  **• Healthy climate (carbon sequestration). • Sea defence. (natural hazard regulation). • Tourism/nature watching. • Clean water and sediments.** | Quantity/Extent | **Composite mapping**: Seagrass extent in the site is 0.52 km² and 99% of this area is within an MPA.  **NE Package:** Conservation objective to “Restore the total extent of seagrass beds to 50.9 ha, and spatial distribution as defined on the map” was not met (Natural England, 2023).  The extents of the majority of subtidal seagrass beds within the study site declined in extent between 2012 and 2018. | **MSFD:** target for all soft substratum habitats (where extent of the habitat is less than 50% of the assessed region). Inside MPAs: extent is stable or increasing (>95% conservation objective 'maintain'. Outside MPAs: area of habitat lost + area of habitat below GES (in condition recover or impacted by unacceptable impact (UoP interpretation LRC 3 or below) ≤ 10% for entire assessed area.  **UK Marine** Strategy**:** Habitat extent shows no loss of extent from previous assessment.  *UoP interpretation Qualitative threshold:* Area in LRC of moderate or below is less than 5% of total habitat extent.  *Quantitative threshold:* Area assessed in condition assessments to be in unfavourable condition is less than 5% of total habitat extent.  *UoP interpretation* Area in LRC of moderate or below is less than 5% of total habitat extent. | Declining (majority of feature) | **C (4)** |
|  |  |  |  | C – last condition assessment 2018 | | C |  |
|  |  |  |  | (2) | | (2) |  |
|  | Subtidal Seagrass | **• Food (Wild Food - fish and shellfish).**  **• Healthy climate (carbon sequestration). • Sea defence. (natural hazard regulation). • Tourism/nature watching. • Clean water and sediments.** | Quality/Condition | **NE Package**: Within the Plymouth Sound and Estuaries SAC, Subtidal Seagrass has a feature condition of “Unfavourable Declining”. Area of subtidal seagrass in ‘unfavourable declining” condition = 47.9% (Natural England, 2023). Condition varied between beds.  The identified adverse conditions likely to be impacting the feature were 1) abrasion, 2) nutrient enrichment and 3) Water clarity.  **LRC:** LRC - 0.23 km^2^ of subtidal seagrass habitat within the PSE SAC site has a LRC score of less than or equal to moderate. This was primarily due to abrasion pressures from recreational anchor and mooring.  **Water Quality:** WQ – in 2019 all 4 water bodies failed to meet WFD standards with a status of ‘moderate’. RNAG for all 4 water bodies included levels of mercury and PBDEs. In addition for Plymouth Sound ‘moderate’ DIN status was a RNAG, however risk of eutrophication from nutrient loading remains low. DO remained at ‘high’ status for all water bodies. | **MSFD**: Pressure based target: extent affected by human disturbance should be minimised, and physical loss is minimised and where possible reversed (Defra, 2019): Inside MPAs: ≥95% of extent to be in favourable condition. i) presence and spatial distribution of biological communities’ representative of the feature are maintained. ii) presence and abundance of key structural and influential species are maintained (≥95% of extent to have conservation objective ‘maintain’). Outside MPAs: as for ‘extent’.  **UK Marine Strategy**: Listed habitats: Area assessed in condition assessments to be in favourable condition with less than 5% unfavourable. If proportion of extent assessed as unfavourable in previous assessment, then this proportion to have decreased.  UoP interpretation  Area in LRC of moderate or below is less than 5% of total habitat extent.  Area in LRC of moderate or below to have reduced since last assessment.  **Water Framework Directive:** WFD Ecological Quality Ratios for seagrass within WFD boundaries.  Water body ecological, chemical and overall condition to be good or better.  DO and DIN levels to meet WFD targets in water body data. | Unfavourable Declining based on data from condition assessments in 2012 and 2018. | **C (4)** |
|  |  |  |  | C – last condition assessment 2018 | | C |  |
|  |  |  |  | (2) | | (2) |  |
|  | Subtidal Seagrass | **• Food (Wild Food - fish and shellfish).**  **• Healthy climate (carbon sequestration). • Sea defence. (natural hazard regulation). • Tourism/nature watching. • Clean water and sediments.** | Spatial configuration | **NE Package:**  **Composite mapping**: Seagrass extent in the site is 0.52 km² and 99% of this area is within an MPA.  **NE Package:** Conservation objective to “Restore the total extent of seagrass beds to 50.9 ha, and spatial distribution as defined on the map” was not met (Natural England, 2023).  The extents of the majority of subtidal seagrass beds within the study site declined in extent between 2012 and 2018.  The conservation objective “Restore the presence and spatial distribution of subtidal seagrass bed communities according to the map” was not met. | As for extent. | As for extent - Declining (majority of feature) | **C (4)** |
|  |  |  |  | C | | C |  |
|  |  |  |  | (2) | | (2) |  |

| **Broad Habitat type** | **Habitat / Species Asset** | **Benefit** | **Characteristic** | **Current Status** | **Target** | **Trend** | **RAG** |
| --- | --- | --- | --- | --- | --- | --- | --- |
| Coastal Margin | Subtidal Seagrass – Firestone Bay | **• Food (Wild Food - fish and shellfish).**  **• Healthy climate (carbon sequestration). • Sea defence. (natural hazard regulation). • Tourism/nature watching. • Clean water and sediments.** | Quantity/Extent | **Composite mapping**: seagrass bed at Firestone Bay to have an area of 0.076 Km^2^.  **NE Package:** The Bunker et al (2019) report estimated a decrease in seagrass area from 7,607 m^2^ to 3,149 m^2^ between 2012 and 2018 at Firestone Bay. Equivalent to an 59% decrease in area. (Natural England, 2023). | **MSFD: As above.**  **UK Marine Strategy: As above.** | Declining  Bunker et al. (2019) report suggests an 59% decrease in seagrass area between 2012 and 2018. | **C (4)** |
|  |  |  |  | C – last condition assessment 2018 | | C |  |
|  |  |  |  | (2) | | (2) |  |
|  | Subtidal Seagrass – Firestone Bay | **• Food (Wild Food - fish and shellfish).**  **• Healthy climate (carbon sequestration). • Sea defence. (natural hazard regulation). • Tourism/nature watching. • Clean water and sediments.** | Quality/Condition | **NE Package**: Average % Seagrass Cover DDV decreased from 21 in 2012 to 17 in 2018.  **LRC:** LRC score above > moderate, due to lack of abrasion pressures.  **Water Quality:** This seagrass bed is within the Plymouth Sound water body area. Water body fails to achieve good status due to levels of chemical contaminants and levels of DIN. Risk of eutrophication is supposedly low due to good-high status of phytoplankton and macroalgae. | **MSFD: As above.**  **UK Marine Strategy: As above.**  **WFD: As above.** | Unfavourable Declining based on data from condition assessments in 2012 and 2018. | **C (4)** |
|  |  |  |  | C – last condition assessment 2018 | | C |  |
|  |  |  |  | (2) | | (2) |  |
|  | Subtidal Seagrass – Firestone Bay | **• Food (Wild Food - fish and shellfish).**  **• Healthy climate (carbon sequestration). • Sea defence. (natural hazard regulation). • Tourism/nature watching. • Clean water and sediments.** | Spatial configuration | **Composite mapping**: seagrass bed at Firestone Bay to have an area of 0.076 Km^2^.  **NE Package:** The Bunker et al (2019) report estimated a decrease in seagrass area from 7,607 m^2^ to 3,149 m^2^ between 2012 and 2018 at Firestone Bay. Equivalent to an 59% decrease in area. (Natural England, 2023). | As for extent. | As for extent - Declining | **C (4)** |
|  |  |  |  | C | | C |  |
|  |  |  |  | (2) | | (2) |  |

| **Broad Habitat type** | **Habitat / Species Asset** | **Benefit** | **Characteristic** | **Current Status** | **Target** | **Trend** | **RAG** |
| --- | --- | --- | --- | --- | --- | --- | --- |
| Coastal Margin | Subtidal Seagrass – Drake’s Island | **• Food (Wild Food - fish and shellfish).**  **• Healthy climate (carbon sequestration). • Sea defence. (natural hazard regulation). • Tourism/nature watching. • Clean water and sediments.** | Quantity/Extent | **Composite mapping**: seagrass bed at Drake’s Island to have an area of 0.052 Km2  **NE Package:** The Bunker et al (2019) report estimated a decrease in seagrass area from 44,207 m^2^ to 40,734 m^2^ between 2012 and 2018 at Drake’s Island. Equivalent to an 8% decrease in area. (Natural England, 2023). | **MSFD: As above.**  **UK Marine Strategy: As above.** | Declining  Bunker et al. (2019) report suggests an 8% decrease in seagrass area between 2012 and 2018. | **C (4)** |
|  |  |  |  | C – last condition assessment 2018 | | C |  |
|  |  |  |  | (2) | | (2) |  |
|  | Subtidal Seagrass – Drake’s Island | **• Food (Wild Food - fish and shellfish).**  **• Healthy climate (carbon sequestration). • Sea defence. (natural hazard regulation). • Tourism/nature watching. • Clean water and sediments.** | Quality/Condition | **NE Package**: Average % Seagrass Cover DDV decreased from 72 in 2012 to 66 in 2018.  Average Shoot Density m^2^ decreased from 97 in 2012 to 64 in 2018. Average Plant Length cm increased from 54 in 2012 to 80 in 2018. % Infected Leaves decreased from 55 in 2012 to 53 in 2018.  **LRC:** Moderate score due to anchor and mooring pressures.  **Water Quality:** This seagrass bed is within the Plymouth Sound water body area. Water body fails to achieve good status due to levels of chemical contaminants and levels of DIN. Risk of eutrophication is supposedly low due to good-high status of phytoplankton and macroalgae. | **MSFD: As above.**  **UK Marine Strategy: As above.**  **WFD: As above.** | Unfavourable Declining based on data from condition assessments in 2012 and 2018. | **C (4)** |
|  |  |  |  | C – last condition assessment 2018 | | C |  |
|  |  |  |  | (2) | | (2) |  |
|  | Subtidal Seagrass – Drake’s Island | **• Food (Wild Food - fish and shellfish).**  **• Healthy climate (carbon sequestration). • Sea defence. (natural hazard regulation). • Tourism/nature watching. • Clean water and sediments.** | Spatial configuration | **Composite mapping**: seagrass bed at Drake’s Island to have an area of 0.052 Km^2^  **NE Package:** The Bunker et al (2019) report estimated a decrease in seagrass area from 44,207 m^2^ to 40,734 m^2^ between 2012 and 2018 at Drake’s Island. Equivalent to an 8% decrease in area. (Natural England, 2023). | As for extent. | As for extent - Declining | **C (4)** |
|  |  |  |  | C | | C |  |
|  |  |  |  | (2) | | (2) |  |

| **Broad Habitat type** | **Habitat / Species Asset** | **Benefit** | **Characteristic** | **Current Status** | **Target** | **Trend** | **RAG** |
| --- | --- | --- | --- | --- | --- | --- | --- |
| Coastal Margin | Subtidal Seagrass – Jennycliff North | **• Food (Wild Food - fish and shellfish).**  **• Healthy climate (carbon sequestration). • Sea defence. (natural hazard regulation). • Tourism/nature watching. • Clean water and sediments.** | Quantity/Extent | **Composite mapping**: Composite mapping shows the seagrass bed at Jennycliff North to have an area of 63 m^2^.  **NE Package:** Summary of losses and gains is not included for the seagrass bed at Jennycliff North as seagrass was only recorded from a single drop and it does not constitute a bed (Bunker et al. 2019)(Natural England, 2023). | **MSFD: As above.**  **UK Marine Strategy: As above.** | Unknown – precautionary approach taken. | **C (4)** |
|  |  |  |  | C – last condition assessment 2018 | | C |  |
|  |  |  |  | (2) | | (2) |  |
|  | Subtidal Seagrass – Jennycliff North | **• Food (Wild Food - fish and shellfish).**  **• Healthy climate (carbon sequestration). • Sea defence. (natural hazard regulation). • Tourism/nature watching. • Clean water and sediments.** | Quality/Condition | **NE Package**: Average % Seagrass Cover DDV decreased from 70 in 2012 to 6 in 2018. Not reliable – based on single drop.  **LRC:** Moderate score due to anchor and mooring pressures.  **Water Quality:** This seagrass bed is within the Plymouth Sound water body area. Water body fails to achieve good status due to levels of chemical contaminants and levels of DIN. Risk of eutrophication is supposedly low due to good-high status of phytoplankton and macroalgae. | **MSFD: As above.**  **UK Marine Strategy: As above.**  **WFD: As above.** | Declining – but based on one drop so not reliable – precautionary approach taken. | **C (4)** |
|  |  |  |  | C – last condition assessment 2018 | | C |  |
|  |  |  |  | (2) | | (2) |  |
|  | Subtidal Seagrass – Jennycliff North | **• Food (Wild Food - fish and shellfish).**  **• Healthy climate (carbon sequestration). • Sea defence. (natural hazard regulation). • Tourism/nature watching. • Clean water and sediments.** | Spatial configuration | **Composite mapping**: Composite mapping shows the seagrass bed at Jennycliff North to have an area of 63 m^2^.  **NE Package:** Summary of losses and gains is not included for the seagrass bed at Jennycliff North as seagrass was only recorded from a single drop and it does not constitute a bed (Bunker et al. 2019)(Natural England, 2023). | As for extent. | Unknown – precautionary approach taken. | **C (4)** |
|  |  |  |  | C | | C |  |
|  |  |  |  | (2) | | (2) |  |

| **Broad Habitat type** | **Habitat / Species Asset** | **Benefit** | **Characteristic** | **Current Status** | **Target** | **Trend** | **RAG** |
| --- | --- | --- | --- | --- | --- | --- | --- |
| Coastal Margin | Subtidal Seagrass – Jennycliff South | **• Food (Wild Food - fish and shellfish).**  **• Healthy climate (carbon sequestration). • Sea defence. (natural hazard regulation). • Tourism/nature watching. • Clean water and sediments.** | Quantity/Extent | **Composite mapping**: Composite mapping shows the seagrass bed at Jennycliff South to have an area of 0.015 km^2^.  **NE Package:** The Bunker et al (2019) report estimated a decrease in seagrass area from 14,378 m^2^ to 6,327 m^2^ between 2012 and 2018 at Jennycliff South. Equivalent to an 56% decrease in area. | **MSFD: As above.**  **UK Marine Strategy: As above.** | Declining  Bunker et al. (2019) report suggests an 56% decrease in seagrass area between 2012 and 2018. | **C (4)** |
|  |  |  |  | C – last condition assessment 2018 | | C |  |
|  |  |  |  | (2) | | (2) |  |
|  | Subtidal Seagrass – Jennycliff South | **• Food (Wild Food - fish and shellfish).**  **• Healthy climate (carbon sequestration). • Sea defence. (natural hazard regulation). • Tourism/nature watching. • Clean water and sediments.** | Quality/Condition | **NE Package**: Average % Seagrass Cover DDV decreased from 21 in 2012 to 14 in 2018.  **LRC:** Score > moderate.  **Water Quality:** This seagrass bed is within the Plymouth Sound water body area. Water body fails to achieve good status due to levels of chemical contaminants and levels of DIN. Risk of eutrophication is supposedly low due to good-high status of phytoplankton and macroalgae. | **MSFD: As above.**  **UK Marine Strategy: As above.**  **WFD: As above.** | Declining  Average % Seagrass Cover DDV decreased from 21 in 2012 to 14 in 2018. | **C (4)** |
|  |  |  |  | C – last condition assessment 2018 | | C |  |
|  |  |  |  | (2) | | (2) |  |
|  | Subtidal Seagrass – Jennycliff South | **• Food (Wild Food - fish and shellfish).**  **• Healthy climate (carbon sequestration). • Sea defence. (natural hazard regulation). • Tourism/nature watching. • Clean water and sediments.** | Spatial configuration | **Composite mapping**: Composite mapping shows the seagrass bed at Jennycliff South to have an area of 0.015 km^2^.  **NE Package:** The Bunker et al (2019) report estimated a decrease in seagrass area from 14,378 m^2^ to 6,327 m^2^ between 2012 and 2018 at Jennycliff South. Equivalent to an 56% decrease in area. | As for extent. | Declining  Bunker et al. (2019) report suggests an 56% decrease in seagrass area between 2012 and 2018. | **C (4)** |
|  |  |  |  | C | | C |  |
|  |  |  |  | (2) | | (2) |  |

| **Broad Habitat type** | **Habitat / Species Asset** | **Benefit** | **Characteristic** | **Current Status** | **Target** | **Trend** | **RAG** |
| --- | --- | --- | --- | --- | --- | --- | --- |
| Coastal Margin | Subtidal Seagrass – Cawsand | **• Food (Wild Food - fish and shellfish).**  **• Healthy climate (carbon sequestration). • Sea defence. (natural hazard regulation). • Tourism/nature watching. • Clean water and sediments.** | Quantity/Extent | **Composite mapping**: Composite mapping shows the seagrass bed at Cawsand Bay to have an area of 0.25 Km2.  **NE Package:** The Bunker et al (2019) report estimated an increase in seagrass area from 119,739 m^2^ to 186,214 m^2^ between 2012 and 2018 at Cawsand Bay. Equivalent to an 56% increase in area. | **MSFD: As above.**  **UK Marine Strategy: As above.** | Increasing  Bunker et al. (2019) report suggests a 56% increase in seagrass area between 2012 and 2018. | **B (4)** |
|  |  |  |  | B – last condition assessment 2018 | | A |  |
|  |  |  |  | (2) | | (2) |  |
|  | Subtidal Seagrass – Cawsand | **• Food (Wild Food - fish and shellfish).**  **• Healthy climate (carbon sequestration). • Sea defence. (natural hazard regulation). • Tourism/nature watching. • Clean water and sediments.** | Quality/Condition | **NE Package**: Average % Seagrass Cover DDV, 2018 – 59. Average Shoot Density m^2^, 2018 – 86. Average Plant Length cm, 2018 – 54. % Infected Leaves, 2018 – 41  **LRC:** Moderate due to anchor and mooring pressures.  **Water Quality:** This seagrass bed is within the Plymouth Sound water body area. Water body fails to achieve good status due to levels of chemical contaminants and levels of DIN. Risk of eutrophication is supposedly low due to good-high status of phytoplankton and macroalgae. | **MSFD: As above.**  **UK Marine Strategy: As above.**  **WFD: As above.** | Declining  Average % Seagrass Cover DDV increased from 30 in 2012 to 59 in 2018.  Average Shoot Density m2 increased from 34 in 2012 to 86 in 2018.  Average Plant Length cm increased from 34 in 2012 to 54 in 2018.  % Infected Leaves decreased from 42 in 2012 to 41 in 2018. | **C (4)** |
|  |  |  |  | C – last condition assessment 2018 | | C |  |
|  |  |  |  | (2) | | (2) |  |
|  | Subtidal Seagrass – Cawsand | **• Food (Wild Food - fish and shellfish).**  **• Healthy climate (carbon sequestration). • Sea defence. (natural hazard regulation). • Tourism/nature watching. • Clean water and sediments.** | Spatial configuration | **Composite mapping**: Composite mapping shows the seagrass bed at Cawsand Bay to have an area of 0.25 Km2.  **NE Package:** The Bunker et al (2019) report estimated an increase in seagrass area from 119,739 m^2^ to 186,214 m^2^ between 2012 and 2018 at Cawsand Bay. Equivalent to an 56% increase in area. | As for extent. | Increasing  Bunker et al. (2019) report suggests a 56% increase in seagrass area between 2012 and 2018. | **B**  **(4)** |
|  |  |  |  | B | | A |  |
|  |  |  |  | (2) | | (2) |  |

| **Broad Habitat type** | **Habitat / Species Asset** | **Benefit** | **Characteristic** | **Current Status** | **Target** | **Trend** | **RAG** |
| --- | --- | --- | --- | --- | --- | --- | --- |
| Coastal Margin | Subtidal Seagrass – Yealm | **• Food (Wild Food - fish and shellfish).**  **• Healthy climate (carbon sequestration). • Sea defence. (natural hazard regulation). • Tourism/nature watching. • Clean water and sediments.** | Quantity/Extent | **Composite mapping**: Composite mapping shows the seagrass beds at Yealm to have a total area of 0.19 Km^2^  **NE Package:** The Bunker et al (2019) report estimated a decrease in seagrass area from 57,009 m^2^ to 47,346 m^2^ between 2012 and 2018 at Cellar’s Cove (Yealm). Equivalent to a 17% decrease in area.  The Bunker et al (2019) report also estimated an increase in seagrass area from 11,447 m^2^ to 11,572 m^2^ between 2012 and 2018 at Red Cove South (Yealm). Equivalent to a 1% increase in area. | **MSFD: As above.**  **UK Marine Strategy: As above.** | Declining  Bunker et al. (2019) report suggests a 17% decrease and a 1% increase at Cellar’s Cove (Yealm) and Red Cove South (Yealm) respectively for seagrass area between 2012 and 2018. | **C (4)** |
|  |  |  |  | C – last condition assessment 2018 | | C |  |
|  |  |  |  | (2) | | (2) |  |
|  | Subtidal Seagrass – Yealm | **• Food (Wild Food - fish and shellfish).**  **• Healthy climate (carbon sequestration). • Sea defence. (natural hazard regulation). • Tourism/nature watching. • Clean water and sediments.** | Quality/Condition | **NE Package**: Between 2012 and 2018, Average % Seagrass Cover DDV decreased from: 74 to 69 (Cellar’s Cove); 80 to 55 (Red Cove South); 77 to 46 (Red Cove North); 19 to 11 (Tomb Rock).  Between 2012 and 2018, Average Shoot Density m^2^ decreased from: 122 to 112 (Cellar’s Cove); 134 to 119 (red Cove South).  Between 2012 and 2018 Average Plant Length cm decreased from 63 to 52 (Cellar’s Cove) and increased from 50 to 56 (Red Cove South).  Between 2012 and 2018, % Infected Leaves increased from 50 to 53 (Cellar’s Cove) and decreased from 56 to 29 (Red Cove South.  **LRC:** Moderate due to anchor and mooring pressures.  **Water Quality:** This seagrass bed is within the Plymouth Sound water body area. Water body fails to achieve good status due to levels of chemical contaminants and levels of DIN. Risk of eutrophication is supposedly low due to good-high status of phytoplankton and macroalgae. | **MSFD: As above.**  **UK Marine Strategy: As above.**  **WFD: As above.** | Declining  Between 2012 and 2018, Average % Seagrass Cover DDV decreased from: 74 to 69 (Cellar’s Cove); 80 to 55 (Red Cove South); 77 to 46 (Red Cove North); 19 to 11 (Tomb Rock).  Between 2012 and 2018, Average Shoot Density m^2^ decreased from: 122 to 112 (Cellar’s Cove); 134 to 119 (red Cove South).  Between 2012 and 2018 Average Plant Length cm decreased from 63 to 52 (Cellar’s Cove) and increased from 50 to 56 (Red Cove South).  Between 2012 and 2018, % Infected Leaves increased from 50 to 53 (Cellar’s Cove) and decreased from 56 to 29 (Red Cove South. | **C (4)** |
|  |  |  |  | C – last condition assessment 2018 | | C |  |
|  |  |  |  | (2) | | (2) |  |
|  | Subtidal Seagrass – Yealm | **• Food (Wild Food - fish and shellfish).**  **• Healthy climate (carbon sequestration). • Sea defence. (natural hazard regulation). • Tourism/nature watching. • Clean water and sediments.** | Spatial configuration | **Composite mapping**: Composite mapping shows the seagrass beds at Yealm to have a total area of 0.19 Km^2^  **NE Package:** The Bunker et al (2019) report estimated a decrease in seagrass area from 57,009 m^2^ to 47,346 m^2^ between 2012 and 2018 at Cellar’s Cove (Yealm). Equivalent to a 17% decrease in area.  The Bunker et al (2019) report also estimated an increase in seagrass area from 11,447 m^2^ to 11,572 m^2^ between 2012 and 2018 at Red Cove South (Yealm). Equivalent to a 1% increase in area. | As for extent. | Declining  Bunker et al. (2019) report suggests a 17% decrease and a 1% increase at Cellar’s Cove (Yealm) and Red Cove South (Yealm) respectively for seagrass area between 2012 and 2018. | **C**  **(4)** |
|  |  |  |  | C | | C |  |
|  |  |  |  | (2) | | (2) |  |
